# Flexible multifunctional platform based on piezoelectric acoustics for human–machine interaction and environmental perception

**DOI:** 10.1038/s41378-022-00402-1

**Published:** 2022-09-14

**Authors:** Qian Zhang, Yong Wang, Dongsheng Li, Jin Xie, Ran Tao, Jingting Luo, Xuewu Dai, Hamdi Torun, Qiang Wu, Wai Pang Ng, Richard Binns, YongQing Fu

**Affiliations:** 1 The State Key Laboratory of Fluid Power and Mechatronic Systems, Zhejiang University, 310027 Hangzhou, China; 2 Faculty of Engineering and Environment, University of Northumbria, Newcastle upon Tyne, NE1 8ST UK; 3 Key Laboratory of 3D Micro/Nano Fabrication and Characterization of Zhejiang Province, School of Engineering, Westlake University, 310024 Hangzhou, China; 4 Key Laboratory of Optoelectronic Devices and Systems of Education Ministry and Guangdong Province, College of Physics and Optoelectronic Engineering, Shenzhen University, 518060 Shenzhen, China

**Keywords:** Electrical and electronic engineering, Physics

## Abstract

Flexible human–machine interfaces show broad prospects for next-generation flexible or wearable electronics compared with their currently available bulky and rigid counterparts. However, compared to their rigid counterparts, most reported flexible devices (e.g., flexible loudspeakers and microphones) show inferior performance, mainly due to the nature of their flexibility. Therefore, it is of great significance to improve their performance by developing and optimizing new materials, structures and design methodologies. In this paper, a flexible acoustic platform based on a zinc oxide (ZnO) thin film on an aluminum foil substrate is developed and optimized; this platform can be applied as a loudspeaker, a microphone, or an ambient sensor depending on the selection of its excitation frequencies. When used as a speaker, the proposed structure shows a high sound pressure level (SPL) of ~90 dB (with a standard deviation of ~3.6 dB), a low total harmonic distortion of ~1.41%, and a uniform directivity (with a standard deviation of ~4 dB). Its normalized SPL is higher than those of similar devices reported in the recent literature. When used as a microphone, the proposed device shows a precision of 98% for speech recognition, and the measured audio signals show a strong similarity to the original audio signals, demonstrating its equivalent performance compared to a rigid commercial microphone. As a flexible sensor, this device shows a high temperature coefficient of frequency of −289 ppm/K and good performance for respiratory monitoring.

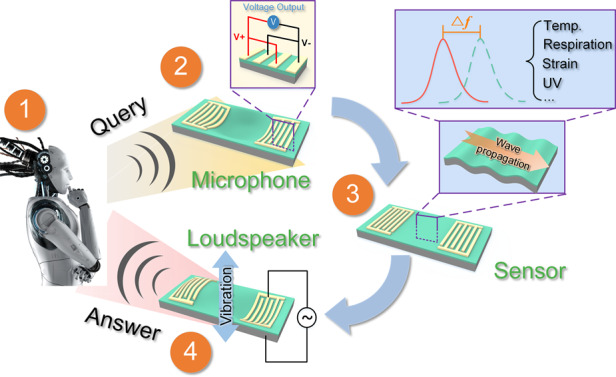

## Introduction

Motivated by great interest and considerable demands, many researchers have recently extensively investigated flexible and wearable electronics, such as flexible transistors^1,2^, flexible thermal interfaces^3^, flexible batteries^4^, flexible acoustic wave devices^5^, flexible displays^6^, point-of-care technology^7^, and wearable products^8^. The rapid development of Internet of Things (IoT) ecosystems^9–12^ also requires soft, compact, reliable, portable and low-power electronics. These integrated flexible platforms with multiple functions can effectively reduce the number of sensing/actuation units and their volumes and/or power consumption.

Most of the existing flexible acoustic wave devices, when applied for applications such as loudspeakers, tend to sacrifice their functional performance in exchange for their flexibility, often resulting in relatively poor performance with less integrated functions compared to their rigid counterparts^5,13^. Flexible acoustic wave devices based on piezoelectric thin films deposited onto flexible substrates are promising solutions for multifunctional, soft, compact and high-performance applications. Electrodes can be conveniently patterned in micro/nanoscales through conventional photolithography, creating various acoustic wave devices that have been adopted for sensing (e.g., temperature^14^, humidity^15^, and cell counting^16^) and actuation applications (e.g., ultrasonic transducers^17^, acoustic heating^18^, and acoustofluidics^19^). The structures and excitation methods of these flexible devices can be flexibly designed and easily achieved, resulting in various vibration modes of acoustic waves. Flexible substrates have much less stiffness than their rigid counterparts (such as Si) and thus are easily deformed under large forces or bending moments. Therefore, acoustic wave devices based on flexible substrates have the potential to be applied as flexible/wearable devices such as microphones and loudspeakers. These devices can be further integrated with human–machine interfaces (HMI) and sensing functions, achieving easy-to-use and compact wearable devices with friendly interfaces. However, most of the reported flexible acoustic devices^20–22^ hitherto are simply modified versions of their rigid counterparts (e.g., by thinning or softening of the substrate) and are often unsuitable for use in wearable applications.

In this paper, we report a flexible acoustic platform based on a zinc oxide (ZnO) thin film deposited on an aluminum (Al) foil substrate, which can easily integrate with acoustic HMI and multiple functions, for applications such as loudspeaker, microphone, and environment perception. Simulations using finite element analysis (FEA) are first performed to investigate the design criteria and optimize the structures of the flexible devices. Comprehensive experiments are then conducted to investigate the performance of the flexible acoustic wave devices. To characterize the flexibility and functionality of the platform as a flexible loudspeaker, properties such as sound pressure level (SPL), directivity, total harmonic distortion (THD), time stability and linearity are studied, and the normalized SPL of the flexible device is then compared with those of rigid ones reported recently in the literature. To evaluate the device’s performance as a microphone, speech recognition experiments are conducted based on the audio signals recorded by the flexible device. By recording a same piece of music, our flexible device’s performance is compared with that of a commercial microphone. The similarity is quantified by the cross-correlation values and the distances determined by dynamic time wrapping (DTW)^23^. Finally, the capacity of this flexible acoustic wave device working as a sensor is demonstrated by measuring the changes of ambient temperatures and monitoring the respiration process.

## Results and discussion

### Working mechanism

**Fig. 1:**
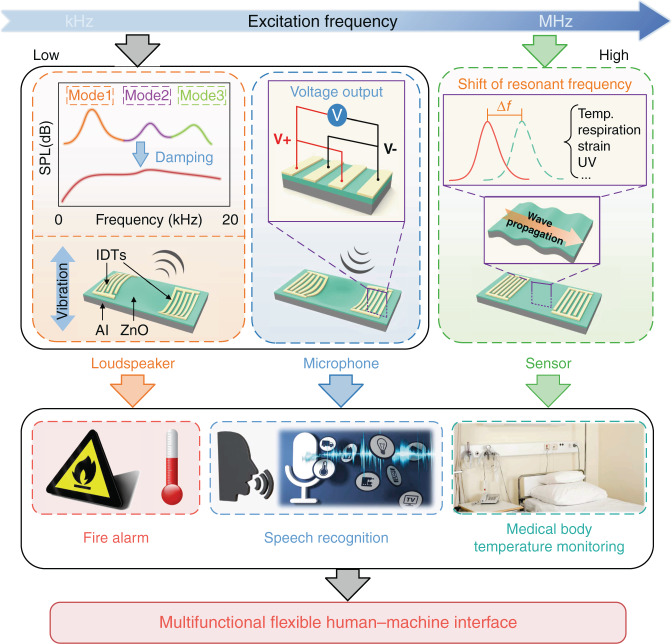
Overall working framework of the flexible platform. Schematic illustrations of the working principle of a flexible ZnO/Al device used as a loudspeaker, microphone and sensor, and its potential applications as a multifunctional human–machine interface

Figure 1 schematically illustrates the working mechanisms of the proposed flexible piezoelectric platform, which contains three different layers. The substrate is Al foil (50 μm thick), on which a piezoelectric ZnO film (~5 μm thick) is deposited. The top layer is the patterned interdigital electrodes (IDEs) composed of Cr (5 nm thick)/Au (80 nm thick). When low-frequency electrical signals (<20 kHz) are applied, periodic electric fields and stresses are produced in the piezoelectric ZnO film due to the piezoelectric effect. As a result, the device vibrates and generates sound pressure with the identical frequency of the electrical signals. In contrast, the foil-structure is forced to vibrate and thus generates a net charge between the IDEs as the low-frequency acoustic signals are received^24^. The original acoustic signals can be reproduced by measuring the voltages on the IDEs caused by the accumulated charges. Working as a flexible acoustic wave sensor, its operating frequency is determined by the wave velocity and the wavelength of the IDE. A high resonant frequency, usually tens of MHz to hundreds of MHz, is applied to generate SAWs, as the increased frequency will enhance the sensitivity of the SAW devices. This principle has been used in the sensing of temperature^25^, respiration^26^, strain^27^, and ultraviolet (UV) radiation^26^. As a highly integrated, high-performance and multifunctional flexible platform, it shows broad prospects in medical applications, body temperature monitoring, respiration or UV sensing, fire alarm and smart wearable devices.

To characterize the design criteria of such a flexible platform, FEA simulations were first conducted for the integrated system. A simplified 3D model was built using COMSOL software to investigate the mechanical vibrations of the flexible device as well as the SPLs it can generate. Detailed information and assumptions can be found in the section “Simulation method” and the supporting information (SI). As similar flexible devices have been proven effective in the excitation of SAWs^16,28^, the main purpose of the optimization using the FEA in this study is focused on improving their performance in low-frequency bands, working as loudspeakers. Because the size of the top electrodes (IDTs) is constrained by the design criteria of the SAW devices, the simulation is carried out based on a rectangular membrane of 32 mm × 21 mm.

#### Optimization of the bottom electrode

**Fig. 2:**
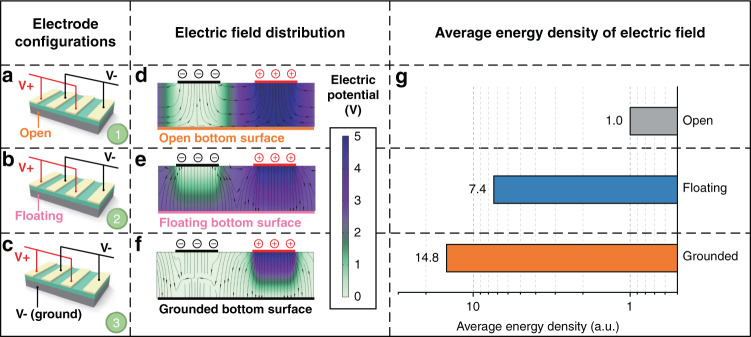
Simulations for investigating the optimization of electrode configurations. **a**–**c** Three electrode configurations with **a** open, **b** floating and **c** grounded bottom surface boundaries. **d**–**f** The simulated electric field distributions corresponding to the three electrode configurations, demonstrating that the bottom electrode can effectively constrain the electric field in (**e**) and (**f**). **g** The simulated average energy densities of the electric field corresponding to the three electrode configurations

For conventional piezoelectric resonators, the function of electrodes is to generate and constrain a specific electric field. The configurations of electrodes determine the distributions of electric fields and excitation forces. Combining the top IDEs and/or bottom electrode, this flexible acoustic wave platform can be electrically powered in three different modes, i.e., open, floating and grounded bottom surface boundaries, as illustrated in Fig. 2a–c.

Figure 2d–f show the simulation results of electric field distributions for the flexible acoustic wave devices with the different electrode configurations in Fig. 2a–c, respectively. The results indicate that the bottom electrode (whether floating or grounded) can effectively constrain the electric field within the piezoelectric layer, resulting in a higher effective electromechanical coupling coefficient and energy conversion efficiency^29,30^.

Figure 2g shows that the devices with floating or grounded bottom electrodes generate higher average electric energy densities in the piezoelectric layer, which are ~7.4 times and ~14.8 times that of the device with an open bottom surface boundary, respectively. As a result, a bottom electrode, whether floating or grounded, is suggested to be included in the configuration for a higher energy conversion efficiency.

#### Substrate effects

**Fig. 3:**
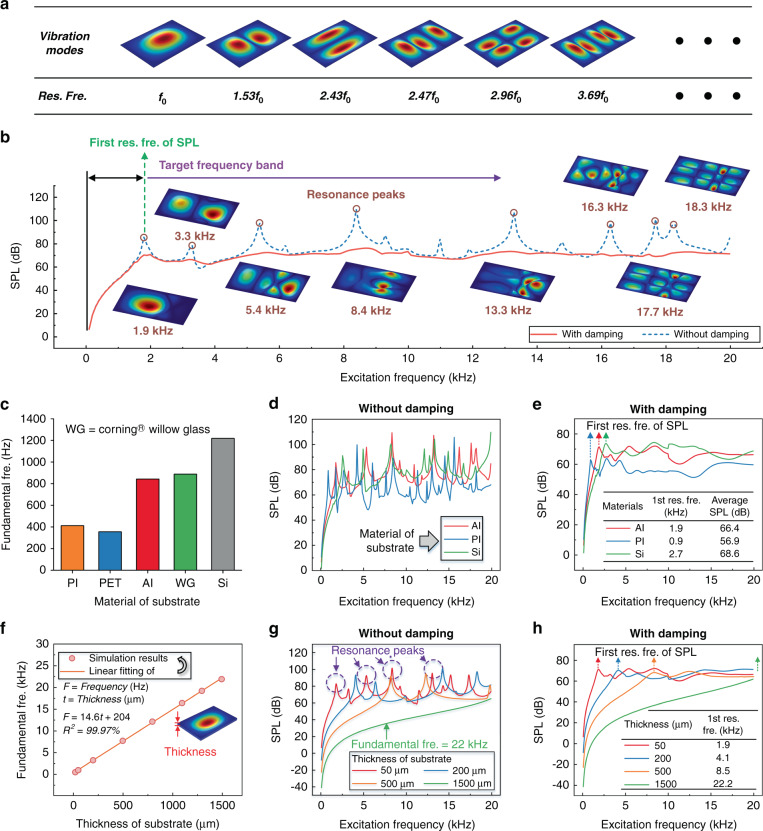
Optimization on the substrate of the flexible device by simulations. **a** First six order out-of-plane vibration modes. **b** The simulated SPLs of the flexible loudspeaker, including a set of resonance peaks determined by its natural mode. The simulation result with damping schematically illustrates the ideal performance, i.e., the SPL reaches a plateau in the frequency band greater than the 1st resonant frequency. Investigations on the effect of substrate materials on **c** fundamental frequencies, **d** SPLs with damping, and **e** SPLs without damping. Investigations on the effect of substrate thickness on **f** fundamental frequencies, **h** SPLs with damping and **h** SPLs without damping, generated by flexible devices using Al substrate with thicknesses of 50, 200, 500, and 1500 μm

**Fig. 4:**
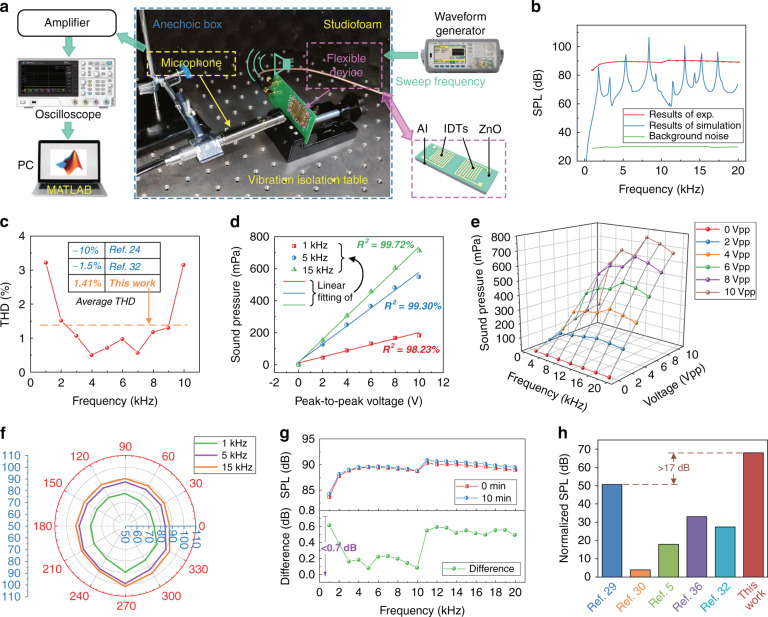
Evaluation of the performance of the flexible device as a loudspeaker. **a** Experimental setup for the measurement of the SPL generated by the flexible loudspeaker at a distance of 5 cm. **b** The experimental and simulated SPLs of the flexible loudspeaker as well as the background noise in the frequency band <20 kHz. **c** The THD of a flexible loudspeaker in the frequency band from 1 to 10 kHz, demonstrating an average THD of ~1.41%, which outperforms other similar works. **d** and **e** The sound pressure generated as a function of excitation voltage, which shows high linearity. **f** The SPL directivity with excitation frequencies of 1, 5, and 15 kHz, demonstrating similar performance in all directions with standard deviations of ~4 dB. **g** Time stability evaluated by the difference between the SPL measured after working for 10 min and the initial state. **h** After normalizing the effect of device area, excitation voltage and measurement distance, the proposed flexible loudspeaker is compared with other similar studies reported in the literature

Figure 3a shows that the flexible acoustic wave device can be approximated as a thin membrane since its lateral dimensions are much larger than its thickness. This membrane can generate a set of out-of-plane resonance modes when its edges are fixed. The SPLs generated by the flexible loudspeaker are estimated using COMSOL simulation, and the detailed procedures can be found in the section “Simulation method” and the SI. Figure 3b shows the obtained results, which contain the resonant peaks corresponding to several specific resonance modes of the flexible device. Flexural deformation and damping of the flexible device as well as the surrounding atmosphere could inhibit the generation of strong resonance peaks. These can be qualitatively verified based on the simulation results shown in Fig. 3b, and subsequently verified experimentally, as shown in Fig. 4b. In the frequency band <20 kHz, the SPLs can be expected to increase first with frequency and then reach a plateau in the higher range of the frequency band (or the targeted frequency band). The starting frequency of the targeted band is mainly determined by the first-order resonant frequency (fundamental frequency) of the out-of-plane modes of the flexible device. By applying the optimized configurations, most of the human hearing sound range (20 Hz–20 kHz) can be covered by the target frequency band, in which the flexible loudspeaker works. The resonant frequency of a membrane can be expressed by the following equation^24^:

1 $$f \propto t\sqrt {\frac{E}{\rho }}$$

where *f*, *t*, *E* and *ρ* are the resonant frequency, thickness, Young’s modulus and density of the membrane, respectively.

To investigate the substrate effects, FEA simulations are performed with five commonly used materials, including polyimide (PI), polyethylene terephthalate (PET), Al, Willow glass and silicon (Si). The condition without a bottom electrode corresponds to the open bottom surface boundary (Fig. 2a), and the condition with a bottom electrode corresponds to the floating or grounded bottom surface boundaries (Fig. 2b and c). The results show that the addition of a bottom electrode can effectively improve the energy conversion efficiency. Therefore, Al foil substrate as the bottom electrode would be superior for this application if compared with other type of substrates.

To compare the mechanical properties (e.g., elastic constant and density) of various substrates, grounded bottom surface boundaries are applied for the piezoelectric layer on different substrate materials. As shown in Fig. 3c, these five substrate materials can be divided into three types according to their fundamental frequencies, namely, (a) polymers including PI and PET (~400 Hz), (b) Al and Willow glass (~800 Hz), and (c) Si (~1200 Hz). Using PI, Al and Si as representatives of the substrate materials, the SPLs generated by the flexible loudspeaker at a distance of 5 cm are simulated with and without the damping effect. The obtained results are shown in Fig. 3d and e. Compared with those on the PI, the results from the devices on the Al substrate show much higher SPLs of ~10 dB in the targeted frequency band but show slightly lower SPLs in the low-frequency band (i.e., <1.3 kHz). On the contrary, compared to those based on Si, the devices using the Al substrate generate higher SPLs in the low-frequency band (i.e., <2.4 kHz) but comparable SPLs in the targeted frequency band. Therefore, the Al substrate performs well from the perspective of mechanical properties compared with the polymer substrates. Based on these results, the subsequent optimizations are carried out using the Al substrate.

Further simulations are performed to determine the optimum thickness of the substrate, and the obtained results are shown in Fig. 3f–h. According to Eq. (1), for a thin rectangular plate whose thickness is much smaller than its lateral dimensions, the fundamental frequency is approximately proportional to the thickness of the substrate (with a linear regression *R*^2^ of 99.97%), as shown in Fig. 3f. Moreover, the SPLs generated by flexible loudspeakers with substrate thicknesses of 50, 200, 500, and 1500 μm are simulated in the frequency band from 100 Hz to 20 kHz, and the obtained results are shown in Fig. 2g and h. It is observed that changing the thickness of the substrate has little effect on the SPLs generated in the targeted frequency band. However, with increasing substrate thickness from 50 to 1500 μm, the first resonant frequency of the SPL-frequency function is increased from 1.9 to 22.2 kHz. In the frequency band less than the fundamental frequency, the vibration of the device is very weak, resulting in low SPLs, as shown in Fig. 2h. With a substrate thickness of 1500 μm, the fundamental frequency of the flexible loudspeaker is ~22 kHz, and the performance in the frequency band below 20 kHz is significantly deteriorated. Although decreasing the thickness of the device below 50 μm might be effective in further reducing the fundamental frequency and improving the low-frequency performance, the stiffness and mechanical properties of the acoustic wave device will be significantly affected^31^. Therefore, Al foil with a thickness of 50 μm is adopted for the fabrication of the flexible acoustic wave devices.

#### Optimization of the top electrode

The recently reported flexible loudspeakers^5,32–38^ generally adopt identical geometries or structures for both bottom and top electrodes, with the piezoelectric layer sandwiched in between. However, this electrode configuration results in an approximately uniform electric field in the piezoelectric medium, and consequently leads to uniformly distributed excitation forces. The resultant force and moment obtained by combining these excitation forces tend to stretch the piezoelectric material in the thickness direction rather than bending it. Most piezoelectric materials and flexible substrate candidates have Young’s moduli larger than several GPa, and are hard to stretch. This leads to relatively low vibration amplitudes and poor performance of the flexible loudspeakers. On the contrary, in our new design, the top electrode of the Al foil-based flexible device covers only part of the top surface of the piezoelectric layer, resulting in a nonuniform electric field and force distribution^39^. Combined with fully fixed edges, the excitation forces can generate an effective moment to bend the flexible device. For a thin membrane, it is much more effective to bend it rather than stretch it. Therefore, the flexible device shows significantly improved vibration amplitudes compared to those using the conventional electrode configuration in simulation (Fig. S1a). As demonstrated by the simulation results shown in Fig. S1b, the device with the top IDE shows a higher SPL of ~76 dB compared with that using identical top and bottom electrodes (~49 dB).

#### Effect of bending deformation

For a severely bent flexible device, the Al substrate may undergo an elastoplastic deformation, which can change its mechanical properties^27^. In particular, the stiffness can be significantly decreased, which may degrade its performance. To investigate the effect of stiffness of substrate on the performance of the flexible loudspeaker, several simulations are conducted by changing the Young’s modulus of the substrate. The fundamental frequencies of the flexible devices and the SPLs generated are simulated and compared using substrates with different Young’s moduli. As shown in Fig. S2a, the fundamental frequency of the flexible loudspeaker is approximately proportional to the square root of the substrate stiffness (with a linear regression *R*^2^ of 99.8%), which is in good agreement with the result obtained using Eq. (1). In the modulus range from 0.07 to 70 GPa, the SPLs generated in the targeted frequency band increase with increasing Young’s modulus, as shown in Fig. S2b. With the hypothesis that Young’s modulus of the Al substrate is significantly reduced from 70 to 7 GPa after severe deformation, the SPL is decreased by only ~10 dB, demonstrating the good robustness of the flexible loudspeakers.

The SPLs generated by the flexible device are further investigated using simulations with bending radii of 10 and −10 cm. The obtained results are shown in Fig. S3 and are compared with those obtained from a flat device. Although the resonant frequencies are shifted with the applied bending strains, the SPLs are hardly affected, demonstrating their good performance under bending conditions.

### Characterization of the flexible loudspeaker

The performance of the flexible device working as a loudspeaker is then evaluated experimentally using the setup shown in Fig. 4a. An arbitrary waveform signal generator is used to generate a sinusoidal signal with a voltage amplitude of 5 V and frequencies varying from 1 to 20 kHz. Excited using the sinusoidal signals, the flexible device generates acoustic vibrations of the same frequency as the source signals, which are measured using a commercial microphone with a distance of 5 cm. The microphone converts the acoustic signals into electrical signals, which are consequently measured using an oscilloscope. To minimize environmental noise, the experiments are performed on a vibration isolation table in an anechoic box. The SPL can be calculated by:

2 $${{{\mathrm{SPL}}}} = 20\log _{10}\left( {\frac{P}{{P_r}}} \right)$$

where *P* and *P*_r_ are the measured sound pressure and reference sound pressure, respectively. In air, the reference sound pressure is generally taken as 20 μPa, which is used in both the experimental calculations and simulations in this paper.

Figure 4b shows the experimental results of frequency responses, which demonstrate that the flexible device can generate a stable SPL output of ~90 dB (with a standard deviation of 3.6 dB) in the wide frequency band of 1–20 kHz. This experimentally obtained value is even better than the simulation result. The total noise, including acoustic noise and electrical noise, is equivalent to ~30 dB, which is much smaller than the SPL produced by the flexible loudspeaker and thus should have little influence on the experimental results. The measurement of SPL is repeated three times, and the obtained results demonstrate that the standard deviations are <0.3 dB, as shown in Fig. S4. The total harmonic distortion or THD can be expressed by the following equation:

3 $${{{\mathrm{THD}}}} = \sqrt {\frac{{\mathop {\sum}\nolimits_{i = 2}^n {A_i^2} }}{{A_1^2}}}$$

where *A*_1_ and *A*_i_ are the root mean square (RMS) amplitudes of the first and *i*th harmonics, respectively. According to the definition, *n* should be infinity. However, in practice, the first a few harmonics are generally taken for the simplicity of calculation. In this paper, we choose *n* = 5. Figure 4c shows that the obtained THDs of the flexible loudspeaker are <3.5% in the frequency band of 1–10 kHz, with an average THD of ~1.41%. The value is better than those of flexible loudspeakers based on poly(vinylidene fluoride) (PVDF)^32^ and electrets^40^.

Figure 4d shows the generated sound pressures measured as a function of the voltage amplitudes applied to the flexible loudspeaker. Excited by a sinusoidal signal with different frequencies of 1, 5, and 15 kHz, the sound pressures generated by the flexible loudspeaker are linearly related to the amplitude of the excitation voltage (0–10 V_pp_), with *R*^2^ values of 98.23%, 99.30% and 99.72%, respectively. Further experiments investigate the sound pressure as a binary function of excitation voltage and frequency, and the obtained results are shown in Fig. 4e. The sound pressure and voltage amplitude maintain a linear relationship in the frequency band from 1 to 20 kHz, with *R*^2^ values all larger than 98%. The SPL directivity of the flexible loudspeaker is demonstrated by the polar plots with excitation frequencies of 1, 5, and 15 kHz, which are shown in Fig. 4f. The results obtained using the other excitation frequencies are shown in Fig. S5. In the targeted frequency band, the SPLs generated by the flexible loudspeaker in all directions show good consistency with a standard deviation of ~4 dB.

Considering that the mechanical vibration of the flexible device may cause changes in its temperature, internal stresses and boundary conditions, the stability of the flexible loudspeaker operated in a continuous operation mode is further studied. The obtained results are shown in Fig. 4g. To ensure that the device can reach thermal equilibrium, the interval between two measurements is taken as 10 min. Compared with the initial values, the SPLs measured after 10 min of operation are slightly increased (with a maximum value of ~0.7 dB), indicating reasonably good stability over time. Figure 4h shows the experimental results of the SPL after normalizing the device area (1 cm^2^), excitation voltage (1 V) and measurement distance (1 cm) for the flexible loudspeaker in this study, as well as those from recent papers in the literature^5,34,35,37,41^. Assuming that the sound pressure is proportional to the device area, excitation voltage and reciprocal of distance, the normalized SPL can be defined as:

4 $${\rm {SPL}}_n = 20\log _{10}\left( {\frac{{1\,{\rm {cm}}^2}}{S} \cdot \frac{{1V}}{E} \cdot \frac{{1\, {\rm {cm}}}}{d}} \right){\rm {SPL}}_m$$

where SPL_n_, SPL_m_, *S*, *E* and *d* are the normalized SPL, the measured SPL, the area of the device, the excitation voltage and the distance from measurement point to loudspeaker, respectively. The results shown in Fig. 4h reveal a normalized SPL value larger than ~17 dB, which is higher than those of the other flexible loudspeakers reported in the literature^5,34,35,37,41^. Due to the large thermal expansion coefficient of the Al substrate, the temperature stability of the flexible loudspeaker is investigated. The SPLs were measured at room temperature (20 °C) and after heating at 60 °C for 10 min. The results are shown in Fig. S6. The SPLs generated by the flexible device are decreased by ~8 dB after heating.

**Fig. 5:**
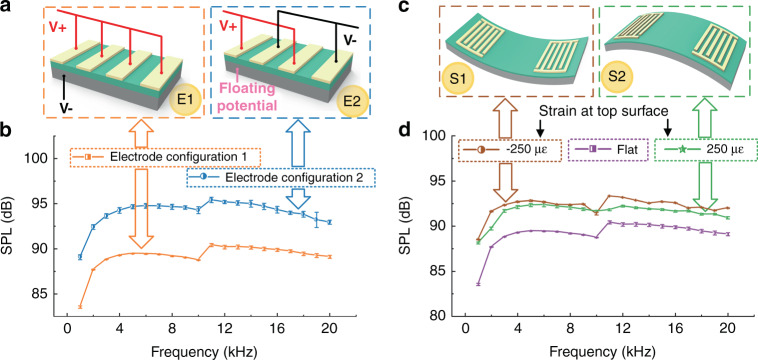
Evaluation of the effects of electrode configuration and bending. **a** and **b** Investigation of the effect of the electrode configuration on the SPL, including **a** two alternatives and **b** experimental results. **c** and **d** Investigation of the performance of the flexible device under bending with radii of 10 cm and −10 cm, respectively. Error bars indicate the standard deviation (*n* = 3)

To investigate the most efficient excitation method for actuating the loudspeaker, the results obtained using two types of electrode configurations are compared, as shown in Fig. 5a. In configuration 1, the top and bottom electrodes have opposite polarities, whereas in configuration 2, the top IDEs are applied with opposite polarities, and the bottom electrode is left with a floating potential. The flexible loudspeaker using configuration 2 shows an SPL value that is ~3 dB higher than that using configuration 1 and thus would be preferred in practical applications.

To study the performance of the flexible loudspeaker under various bending conditions, the SPLs are measured with bending radii of 10 cm and −10 cm, and the obtained results are shown in Fig. 5c and d. The center of the bent loudspeaker is always kept 5 cm away from the microphone during the measurements. As shown in Fig. 5d, the experimental results demonstrate that the SPL of the flexible loudspeaker is hardly affected by bending, regardless of the bending direction.

In brief, the above results clearly show that the flexible device can effectively generate acoustic signals depending on the electrical excitations. As a demonstration of its successful application, an excerpt from Chopin’s Fantaisie-Impromptu is successfully played using our newly developed flexible loudspeaker (see Supplementary Movie 1). The device has been verified to exhibit high performance in terms of SPL, THD, linearity, directivity, frequency stability and time stability when used as a loudspeaker, based on which the flexible HMI can be developed.

### Characterization of the flexible microphone

**Fig. 6:**
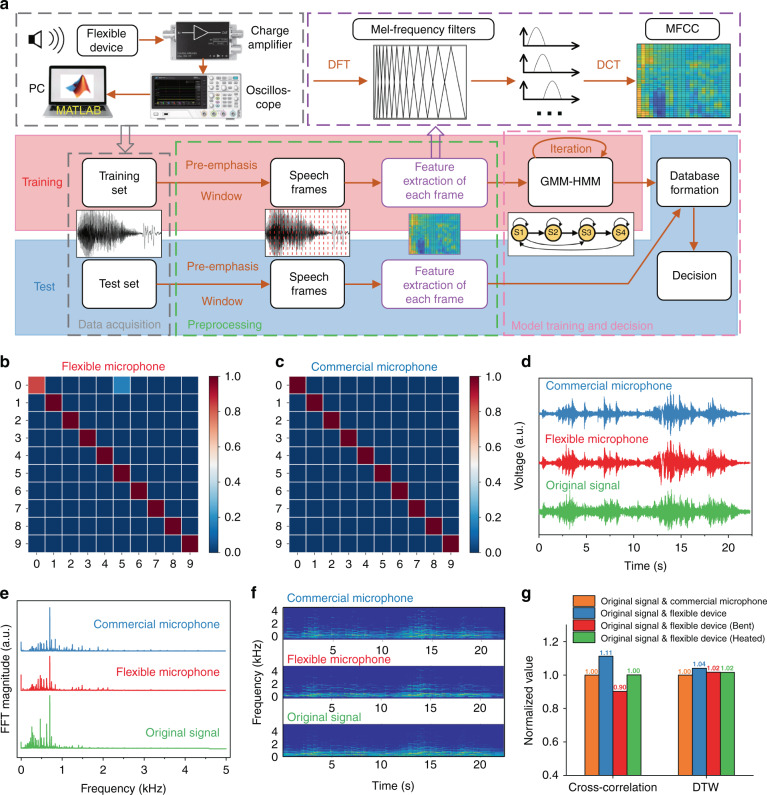
Evaluation of the performance of the flexible device working as a microphone. **a** Flowchart of speech recognition, including data acquisition, preprocessing, model training and decision-making. **b** and **c** Heatmaps indicating the results of speech recognition based on **b** the flexible microphone and **c** a commercial microphone, respectively. **d**–**f** A piece of music as well as the signals recorded by the flexible microphone and the commercial microphone, which are shown in the d time domain, **e** frequency domain, and **f** time–frequency domain. **g** The similarity of the original audio signal and recorded signal in the time domain, which is quantitatively evaluated by the cross-correlation and the distance calculated by the DTW algorithm

To explore the flexible device as a microphone and evaluate its performance, experiments of speech recognition on numbers 0–9 are carried out based on the Gaussian mixture-hidden Markov model (GMM-HMM)^41–45^. The HMM is a mature model for speech recognition, and its effectiveness has been widely proven. Therefore, this algorithm is adopted to prove the effectiveness of the device for speech recognition without the support of any special algorithm. As shown in Fig. 6a, the voltages generated by the flexible microphone are first amplified using a charge amplifier, then measured using an oscilloscope and finally analyzed using a MATLAB program. The collected speech data are divided into a training set and a test set. After preprocessing, speech frames are obtained, and Mel-frequency cepstral coefficients (MFCCs) are extracted for each frame^42^. The GMM-HMM model is trained based on the MFCC, and the performance of the model is verified using the test set^43–45^. All the details of these procedures can be found in the SI.

Figure 6b and c show the results of speech recognition (numbers 0–9) based on the data recorded using both our flexible microphone and a commercial microphone, and their corresponding precision values are 98% and 100%, respectively. This clearly shows that the performance of the proposed flexible microphone is comparable to the corresponding rigid commercial products when applied for speech recognition. To investigate the effect of bending (with a radius of 10 cm) and temperature (heating at 60 °C for 10 min) on the performance of the proposed flexible microphone, the speech recognition experiments are repeated in both cases. The obtained results of precision values, which are almost unaffected, are 97.33% (bending) and 100% (heating), as shown in Fig. S7.

The above experiments demonstrate that the effective features can be extracted from the data recorded by the flexible microphone and can be adopted in the field of speech recognition. However, it is still unclear if the recorded sound information is completely represented or if it can represent the original audio. To verify these results, a piece of music is measured using a flexible microphone and a commercial microphone. The results shown in Fig. 6d reveal that both the obtained results are comparable with the original music in the time domain after normalization. Moreover, these three audio signals are also comparable in the frequency domain and in the time–frequency domain, as clearly shown in Fig. 6e and f, respectively. It can be confirmed that the waveforms recorded by the flexible microphone and the commercial microphone are approximately identical.

To further quantitatively evaluate the performance of the flexible microphone, the maximum value of the cross-correlation function and the distance determined by dynamic time wrapping (DTW)^23^ are calculated to evaluate the similarity of the original signal and the signal recorded by the two microphones in the time domain. A larger maximum value of the cross-correlation function and a smaller value of DTW indicate that the two signals used in the calculation have a better similarity. Figure 6g clearly shows that the maximum value of the cross-correlation function between the signal recorded by the proposed flexible device and the original signal is 11% larger than that between the signal recorded by the commercial microphone and the original signal. The result demonstrates that compared with the commercial product, our proposed flexible device can record with a better similarity to the original signal. The values obtained by the DTW algorithm show slightly different results. The value of DTW between the signal recorded by our proposed flexible device and the original signal is ~4% larger than that between the signal recorded by the commercial microphone and the original signal. The differences in the values are mainly because different algorithms have different definitions of “similarity”. These two methods evaluate the similarity of readings from two different perspectives; therefore, their results are not completely consistent. However, given that the DTW value of the proposed flexible device is only 4% different from that of the commercial microphone, quite good performance is demonstrated. We can confidently conclude that the performance of the flexible microphone is roughly equivalent to that of a commercial rigid counterpart from the perspective of audio recording and reproduction. The effects of bending (radius of 10 cm) and temperature (heating at 60 °C for 10 min) on the performance of the proposed microphone are also investigated, and the obtained results show that neither bending nor heating significantly degrades its performance, as shown in Fig. 6g.

### Performance of the flexible Lamb wave sensor

When high-frequency signals are applied to IDEs, acoustic waves can be excited and then propagate along the surface of the flexible device. The resonant frequency of the acoustic wave is quite sensitive to ambient temperature and mass loading on the device surface, making it suitable for sensing applications. Since the thickness of the flexible acoustic wave device is smaller than the IDT period, the vibration mode is a Lamb wave (or flexural wave).

**Fig. 7:**
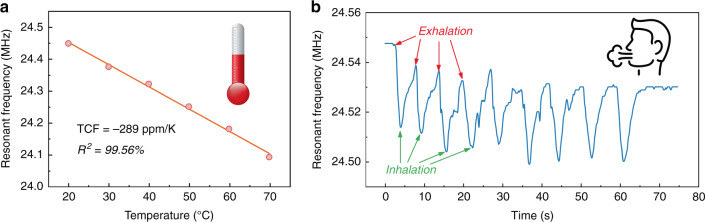
Performance of the flexible Lamb wave sensor. **a** TCF of the A_0_ mode of the flexible device. **b** Variations in the resonant frequency when respiration is measured with the flexible device

Figure 7a shows the measured temperature coefficient of frequency (TCF) values of the SAW devices. The A_0_ mode of a flexible acoustic device with a wavelength of 100 μm presents a high TCF of −289 ppm/K (*R*^2^ = 99.56%), which is applicable for temperature monitoring^25^, such as body temperature monitoring, fire alarm and other similar applications. Furthermore, the flexible device can be implemented for breathing detection/monitoring, and the obtained results are shown in Fig. 7b. The exhaled air will cause variations in both temperature and humidity, thus resulting in an apparent shift in the resonant frequency. The contribution of humidity can be attributed to the variations in mass loadings and electrical impedances due to adsorption and condensation of water molecules. As a flexible device, it can also measure strain <200 με, which has been previously reported in ref. ^27^. Combined with the additional sensing layer, the device can measure other environmental parameters, such as ultraviolet radiation and humidity, which have been previously explored, e.g., ref. ^26^. However, these flexible devices are sensitive to a variety of excitations (especially temperature and humidity levels), which may lead to coupling in the response. To solve this problem, some machine learning methods can be used for data processing^46^. For SAW devices with multiple vibration modes, self-temperature calibration can be achieved by utilizing the different sensitivities of each mode^3^. When the flexible device works as a microphone or loudspeaker, the potential and stress in the piezoelectric layer are forced to change, thus affecting the propagation of the SAWs and the continuous measurement results. Therefore, different functions (microphone/speaker/SAW sensor) are not activated simultaneously. To coordinate the three functions, it is necessary to implement the switching between functions by utilizing control circuits in practical applications. In summary, as a sensing element, the flexible device is applicable to a wide range of situations and shows excellent performance.

## Conclusions

In summary, we developed a flexible multifunctional acoustic platform with good performance. In addition to the generally studied sensing applications, it can work perfectly as a loudspeaker and a microphone through reasonable structural optimization, both of which exhibit excellent performance. As a loudspeaker, the device shows a stable average SPL of ~90 dB (with a standard deviation of 3.6 dB) in the frequency band from 1 to 20 kHz with a measurement distance of 5 cm and an excitation voltage of 5 V. Evaluated by the SPL normalized by device area, excitation voltage and measurement distance, the proposed flexible device in this study outperforms the flexible loudspeakers recently reported in the literature. In addition, the SPL of the device shows a low THD (average value of ~1.41% from 1 to 10 kHz), uniform directivity (with a standard deviation of ~4 dB), good stability over time, and high linearity to excitation voltage (*R*^2^ > 98%). The performance of the device as a microphone is further characterized by speech recognition and audio recording. The precision of speech recognition based on the flexible microphone is 98%, which is comparable to that based on the commercial microphone. To determine the similarity between the original audio signal and the signal recorded by a microphone, the maximum value of the cross-correlation function is calculated, and the flexible microphone performs a higher value of ~11% than the commercial microphone, reflecting its excellent fidelity for acoustic recording. Combining the capacity of interaction with the sensing functions, the applications of the flexible SAW device can be greatly broadened. For example, it is possible to be activated and set up through voice commands, then conduct corresponding sensing work, and report the results through voice. As a high-performance HMI with versatility, the flexible device is promising and competitive for integration into next-generation health monitoring, wearable, and intelligent medical systems.

## Experimental section

### Fabrication of the flexible acoustic device

For fabrication of the flexible device, an Al foil with a thickness of ~50 μm was applied as the flexible substrate. A ZnO thin film of ~5 μm was then deposited onto the Al foil using DC magnetron sputtering technology. During deposition, the Ar/O_2_ gas flow rate was set as 10/13 sccm, and the DC target power was set as 400 W. A zinc target of 99.99% purity was kept 70 mm away from the sample, and the chamber pressure was ∼3 mTorr. The IDTs, composed of Cr (5 nm thick)/Au (80 nm thick), were fabricated onto the ZnO thin film using the standard photolithography and lift-off technique.

### Experimental setup and characterization

To evaluate the flexible device as a loudspeaker, the flexible device was taped onto a printed circuit board (PCB), which can be approximately regarded as a constraint of fully fixed edges. To ensure the repeatability of the bonding, the flexible device was removed from and then taped onto the PCB three times, and the SPLs were measured. The results are shown in Fig. S8, demonstrating that the performance shows good repeatability. The input signal was generated by an arbitrary waveform generator (Agilent 33522A) with an amplitude of 5 V and frequencies from 1 to 20 kHz. The sound pressure was measured using a commercial microphone (AWA14403) and a preamplifier (AWA14600E) with a sensitivity of 15 mV/Pa, which was placed 5 cm away from the flexible loudspeaker. The output voltage was measured and recorded using an oscilloscope (ZLG ZDS1104), which was then preprocessed and analyzed using a MATLAB program. In addition, the reference sound pressure was taken as 20 μPa.

To investigate the performance of the loudspeaker under bending conditions, a flexible printed circuit board (FPC) was designed and manufactured to replace the PCB. The flexible device and FPC were taped on a cylindrical resin surface prepared by 3D printing with a radius of 10 cm. The other experimental details are identical to those in the flat condition.

To evaluate the flexible device working as a microphone, the voltage measured on the IDEs of the flexible device was first amplified using a charge amplifier (Femto HQA-15M-10T) and then measured and recorded using an oscilloscope. The following speech recognition, including preprocessing of data, model training and decision, were conducted using a MATLAB program. The details of the speech recognition experiments can be found in the SI.

### Simulation method

To investigate the vibration modes and SPLs of the flexible loudspeaker, FEA simulations were performed using COMSOL software. A simplified three-dimensional (3D) model comprising double layers was formulated, i.e., a ZnO (thickness of 5 μm) thin film on an Al substrate (with thicknesses of 50, 200, 500, or 1500 μm). The mass loading effect of the IDEs was neglected in analyzing the low-frequency vibrations because of their relatively small thickness compared to the other two layers. Therefore, the top electrodes were not incorporated in the module of solid mechanics and were simplified and characterized by applying proper electrical boundary conditions on the corresponding region of the ZnO surface. This rectangular membrane (dimensions of 32 mm × 21 mm thickness) was excited by a voltage with an amplitude of 5 V and was mechanically constrained by fully fixed edges.

### Associated content

#### Supporting information

Simulated average vibration amplitudes and SPLs of the flexible loudspeakers with two different top electrode configurations; effect of substrate stiffness on the properties of the flexible loudspeaker investigated by simulation; photos and simulated SPLs of the flexible loudspeaker under bending conditions; directivity of SPL of the flexible device excited by electrical signal with frequencies of <20 kHz; theoretical assumptions about bending; details about speech recognition process; audio excerpt from Chopin’s Fantaisie-Impromptu played using the flexible loudspeaker.

## Supplementary information

Supporting Information

Supplementary Movie 1
