# Supplementary material for: Flexible multifunctional platform based on piezoelectric acoustics for human–machine interaction and environmental perception
Source: Microsyst Nanoeng. 2022 Sep 14;8:99. doi: 10.1038/s41378-022-00402-1 (PMC9474866; doi:10.1038/s41378-022-00402-1)
Supplement: Supplementary file 1 — Supporting Information [file 41378_2022_402_MOESM1_ESM.docx]

Supporting Information

**Flexible Multifunctional Platform Based on Piezoelectric Acoustics for Human-Machine Interaction and Environmental Perception**

Qian Zhang^a,b^, Yong Wang^a,c^, Dongsheng Li^a^, Jin Xie^a,^*, Ran Tao^d^, Jingting Luo^d^, Xuewu Dai^b^, Hamdi Torun^b^, Qiang Wu^b^, Wai Pang Ng^b^, Richard Binns^b^, and YongQing Fu^b,a,^*

^a^ The State Key Laboratory of Fluid Power and Mechatronic Systems, Zhejiang University, Hangzhou 310027, China

^b^ Faculty of Engineering and Environment, University of Northumbria, Newcastle upon Tyne NE1 8ST, UK

^c^ Key Laboratory of 3D Micro/Nano Fabrication and Characterization of Zhejiang Province, School of Engineering, Westlake University, Hangzhou 310024, China

^d^ Key Laboratory of Optoelectronic Devices and Systems of Education Ministry and Guangdong Province, College of Physics and Optoelectronic Engineering, Shenzhen University 518060, China

* Corresponding authors: xiejin@zju.edu.cn; richard.fu@northumbria.ac.uk

**Figures**


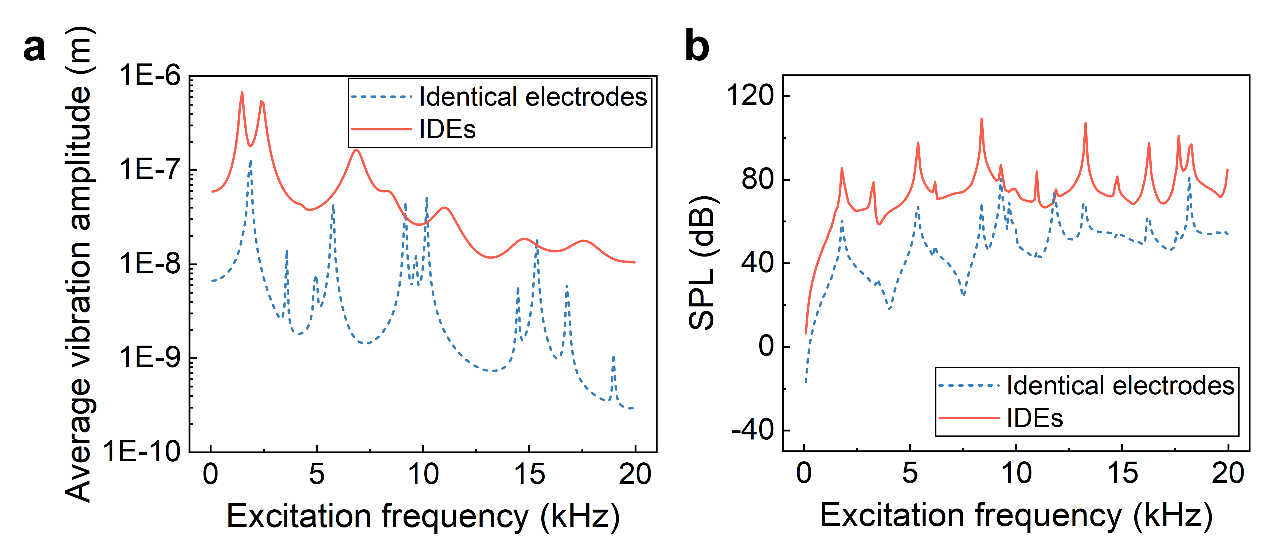


**Figure S1.** The simulated a) average vibration amplitudes; and b) sound pressure levels (SPLs) of the flexible loudspeakers with two different top electrode configurations, i.e., a fully covered top electrode, and an interdigital electrode (IDE).


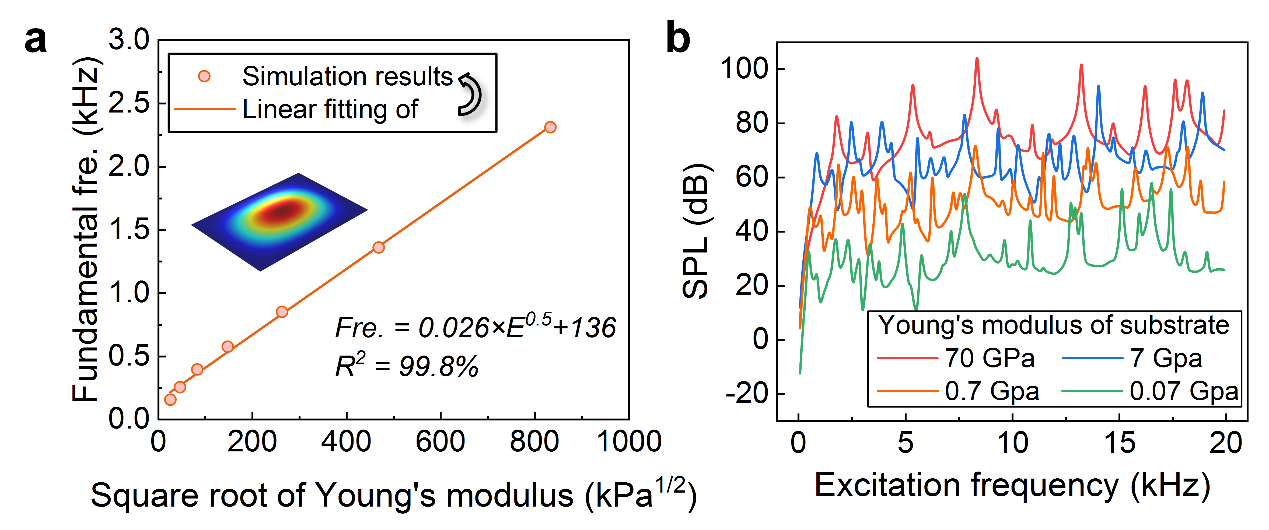


**Figure S2.** Effect of substrate stiffness on properties of flexible loudspeaker investigated by simulation. a) The fundamental frequency as a function of the square root of Young’s modulus of the substrate. b) The SPLs produced by flexible devices with substrate’s Young’s modulus varied from 0.07 GPa to 70 GPa.

**Discussion about Figure S2b**

Intuitively, a large substrate stiffness tends to cause a small vibration amplitude and a small SPL, which is exactly opposite to the simulation results, as shown in Figure S2b. This is because for a double-layered thin membrane such as the proposed flexible device in this study, the normal displacement mainly comes from the bending moment caused by the difference between the lateral deformations of piezoelectric layer and substrate. The magnitude of this normal displacement is significantly affected by the position of neutral layer. However, changing the substrate’s stiffness will result in a variation of the position of the neutral layer. With an assumption that the substrate stiffness is much less than that of the piezoelectric material, the neutral layer will be in the piezoelectric layer, resulting in small bending moment, small vibration amplitude and low SPL.


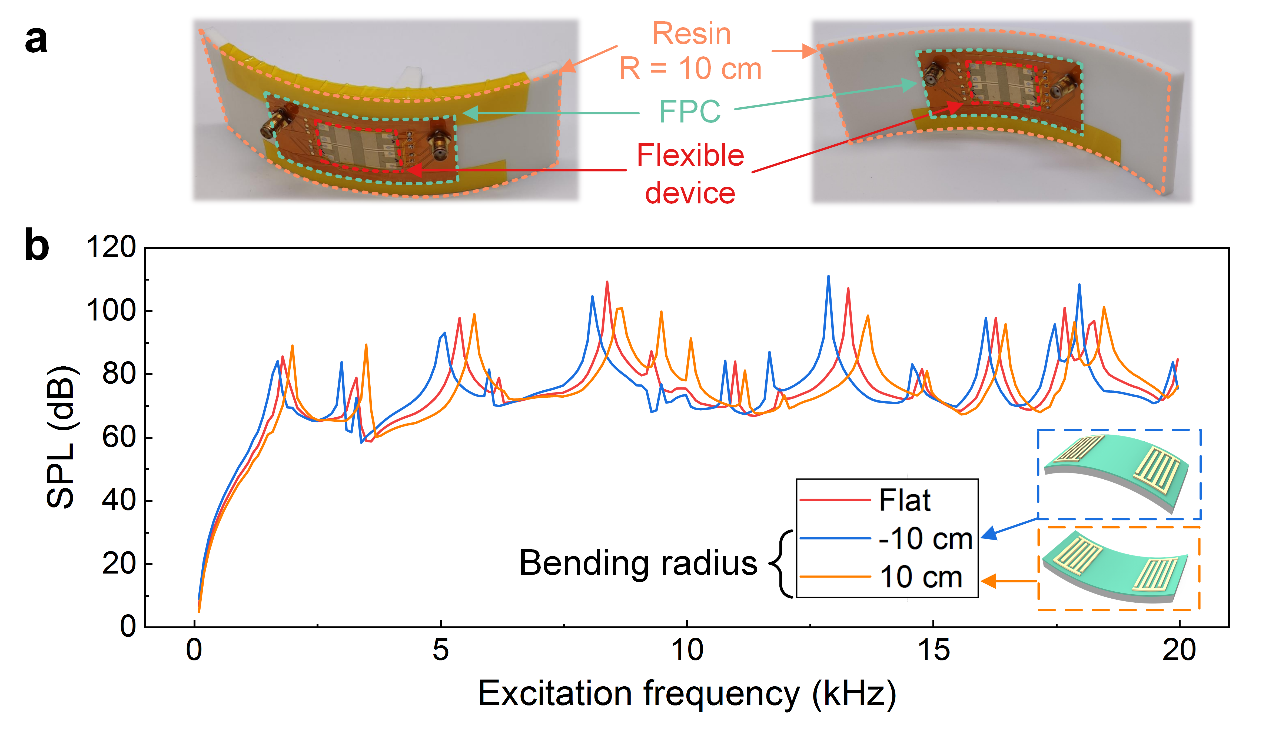


**Figure S3.** a) Photos of experimental setup to bend the flexible loudspeaker with radii of 10 cm and -10 cm. b) Simulated SPLs of flat and bent flexible devices with an excitation voltage of 5 V and a measurement distance of 5 cm.

**Figure S4.** The repeatability of the SPL of the proposed device as a loudspeaker. Error bars indicate the standard deviation (n = 3).

**Figure S5.** The directivity of SPL of the flexible device excited by electrical signals with frequencies of less than 20 kHz.

**Figure S6.** Temperature stability of the proposed flexible loudspeaker, revealing from the SPL signals measured at 20 ^o^C and that measured after heating at 60 ^o^C for 10 minutes.


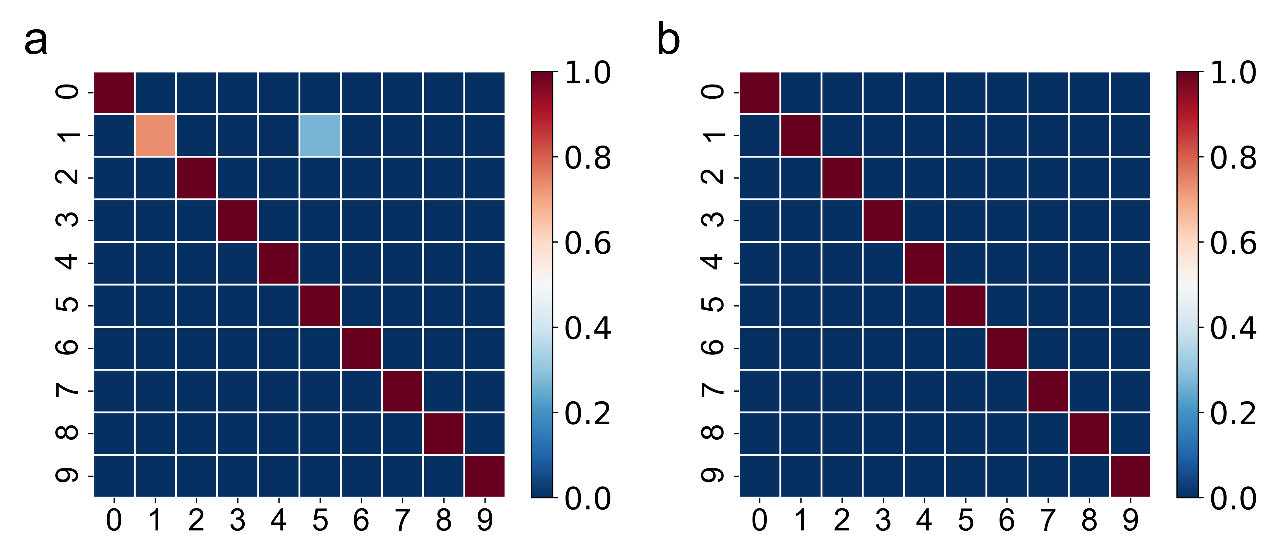


**Figure S7.** Heat map indicating the results of speech recognition based on (a) bent and (b) heated flexible devices, respectively. For the bent device, the bending radius is 10 cm. For the heated device, the heating is conducted at 60 ^o^C for 10 minutes.

**Figure S8.** The SPLs of the proposed flexible device measured for three times. The proposed device is manually removed and pasted again before each measurement. Error bars indicate the standard deviation (n = 3).

**Assumptions about bending**

To analyze and simulate the device under bending, some assumptions are proposed to simplify the model. The device is assumed under a pure bending, and the strain is proportional to the distance to the neutral layer. Because the thickness of the substrate is much larger than that of the piezoelectric layer, only the change of mechanical properties of the substrate is considered. Strain will lead to the variation of material density, which are described by equation [1]:

$\Delta\rho/\rho=-\left(\varepsilon_{1}+\varepsilon_{2}+\varepsilon_{3}\right)$ (S1)

where $\text{ε}_{\text{1}}$, $\text{ε}_{\text{2}}$, $\text{ε}_{\text{3}}$ are the strains in three orthogonal directions. The strain will lead to the variations of material stiffness and introduce internal stress, which approximately takes the form [2]:

$\varepsilon\left(\sigma\right)=\sigma/E+\varepsilon_{yield}(\sigma/\sigma_{yield})^{n}$ (S2)

where *σ*, *E*, *ε_yield_*, *σ_yield_*, *n* are the stress, the Young’s modulus without strain, the strain corresponding to yield strength, the yield strength and a constant determined by material properties, respectively. The Young’s modulus under strain can be approximated as the slope, i.e., ${\text{d}\text{σ}}/{\text{d}\text{ε}}$. The above modifications are introduced into the finite element analysis FEA simulation model to approximate the calculation of the SPL produced by the flexible loudspeaker under the bending.

**Speech recognition**

A speech recognition experiment on ten numbers (from 0 to 9) was performed based on the voice data recorded by the flexible microphone and a commercial microphone. To effectively conduct the speech recognition, the mel-frequency cepstral coefficient (MFCC) was extracted first, which were used as features to describe the phoneme contained in the voice signal in this paper [3]. To improve the signal-to-noise ratio (SNR) in the high frequency band, pre-emphasis was conducted using a filter, which can be expressed as [4]:

$H\left(z\right)=1-\mu z^{-1}$ (S3)

where *μ* is taken as 0.9375. The continuous signal was separated into frames of 20 ms with 10 ms overlapped between adjacent frames on the hypothesis that the voice system is stationary in each frame. With a sampling frequency of 50 kHz, the length of frame is 1000 points. The power spectrum of each frame was estimated of by discrete Fourier transform (DFT), which can be expressed as:

$\begin{matrix} V\left(k\right)=\sum_{n=1}^{N} v\left(n\right)w\left(n\right)e^{-j2\pi kn/N},1\leq k\leq N \\ P\left(k\right)=\frac{\left|V\left(k\right)\right|^{2}}{N},1\leq k\leq N \end{matrix}$ (S4)

where *v* and *V* are the voice signals in time and frequency domain, respectively, *w* is hamming window of *N* points, *N* is taken as 1000 and *P* is the estimation of power spectrum.

To match the characteristics of human auditory, 24 overlapped triangular filters in mel-domain were used to calculate the log-energy of the *P* passing through each filter. The mel-scale and its inverse are defined as [4]:

$\begin{matrix} M\left(f\right)=1125\ln\left(1+\frac{f}{700}\right) \\ M^{-1}\left(m\right)=700\left(e^{m/1125}-1\right) \end{matrix}$ (S5)

where *M* is the mel-scale frequency, *f* is the linear frequency. A group of frequencies uniformly spaced in mel-domain is defined as [4]:

$f\left[m\right]=\left(\frac{N}{F_{s}}\right)M^{-1}\left(M\left(f_{l}\right)+a\frac{M\left(f_{h}\right)-M\left(f_{l}\right)}{A+1}\right),a=1,2,...,A$ (S6)

where *N* is the length of FFT, *F_s_* is the sampling rate in Hz, *A* is the number of filters, *f_l_* and *f_h_* are the starting and ending frequencies of the filterbank. According to Ref. [4], the filterbanks can be expressed as:

$H_{m}\left(k\right)=\left\{\begin{matrix} 0 & k<f\left[m-1\right] \\ \frac{k-f\left[m-1\right]}{f\left[m\right]-f\left[m-1\right]} & f\left[m-1\right]\leq k\leq f\left[m\right] \\ \frac{f\left(m+1\right)-k}{f\left[m+1\right]-f\left[m\right]} & f\left[m\right]\leq k\leq f\left[m+1\right] \\ 0 & k>f\left[m+1\right] \end{matrix}\right.$ (S7)

Subsequently, the DCT of these 24 log-energies are calculated, generating the envelope of the signal called cepstral coefficients. The MFCC generally takes the second to thirteenth cepstrum coefficients. To describe the dynamics of the signal, delta coefficients are also involved, which is calculated by the five-points regression formula:

$d_{t}=\frac{\sum_{n=1}^{N} n\left(c_{t+n}-c_{t-n}\right)}{2\sum_{n=1}^{N} n^{2}}$ (S8)

where *d_t_* is the delta coefficients, *N* is taken as 2, *c* is the coefficients of a certain frame and *t* is the number of frames.

To match the transition of phonemes, a hidden Markov model (HMM) with 4 hidden states is built, each of which corresponds to a Gaussian mixture model with 3 components. For each number, the Baum-Welch method [5] is used to estimate the parameters of individual GMM-HMM, including the mean value, covariance and the weight of each Gaussian component, and the state transition matrix of the HMM. For numbers from 0 to 9, 8 training samples and 40 iterations are adopted for each number, which is sufficient to reach convergence.

For recognition of an unlabeled word, the prepared 10 GMM-HMM are scored depending on the possibility of generating the observation sequence (the MFCC features of the word). The number corresponding to the model with the highest score is taken as the identification result. For each number, there are about 10 voice signals in the test set.

**References**

[1] Nalamwar, A. L. & Epstein, M. Surface acoustic waves in strained media. *J. Appl. Phys.* **47**, 43–48 (1976).

[2] Ramberg, W. & Osgood, W. R. Description of stress-strain curves by three parameters. *Natl. Advis. Comm. Aeronaut.* Technical Note No. 902 (1943).

[3] Davis, S. & Mermelstein, P. Comparison of parametric representations for monosyllabic word recognition in continuously spoken sentences. *IEEE Trans. Acoust.* **28**, 357–366 (1980).

[4] Huang, X., Acero, A., Hon, H.-W. & Reddy, R. Spoken language processing: A guide to theory, algorithm, and system development. (Prentice hall PTR, 2001).

[5] Rabiner, L. R. A Tutorial on Hidden Markov Models and Selected Applications in Speech Recognition. *Proc. IEEE* **77**, 257–286 (1989).
